# Unraveling Band‐Tail Effects on Temperature‐Dependent Emission in GaAsBi via Photoluminescence

**DOI:** 10.1002/advs.202516349

**Published:** 2025-11-30

**Authors:** Bing Yan, Xiren Chen, Liangqing Zhu, Lijuan Wang, Man Wang, Shumin Wang, Jun Shao

**Affiliations:** ^1^ School of Physics and Engineering Henan University of Science and Technology Luoyang 471023 China; ^2^ National Key Laboratory of Infrared Detection Technologies Shanghai Institute of Technical Physics Chinese Academy of Sciences Shanghai 200083 China; ^3^ Key Laboratory of Infrared Imaging Material and Detectors Shanghai Institute of Technical Physics Chinese Academy of Sciences Shanghai 200083 China; ^4^ Engineering Research Center for Nanophotonics and Advanced Instrument Ministry of Education East China Normal University Shanghai 200062 China; ^5^ State Key Laboratory of Functional Materials for Informatics Shanghai Institute of Microsystem and Information Technology Chinese Academy of Sciences Shanghai 200050 China; ^6^ State Key Laboratory of Infrared Physics Shanghai Institute of Technical Physics Chinese Academy of Sciences Shanghai 200083 China; ^7^ Photonic Laboratory Department of Microtechnology and Nanoscience Chalmers University of Technology Göteborg S‐412 96 Sweden; ^8^ Hangzhou Institute for Advanced Study University of Chinese Academy of Sciences Hangzhou 310024 China

**Keywords:** band‐tail states, GaAsBi, photoluminescence, temperature sensitivity

## Abstract

The effect of Bi on the emission temperature sensitivity of GaAsBi remains a topic of debate, which hinders the design of optoelectronic devices. Band‐tail states, which are critical for GaAsBi performance, are suspected to drive the discrepancy, but their effect remains unclear. This work resolves the key debate using an innovative dual‐spectroscopy approach that combines temperature‐dependent photoluminescence (PL) and transmission spectroscopy to decouple the contributions of band‐tail states from intrinsic band‐edge behavior. For GaAs_1‐_ *_x_* Bi*_x_* (*x *= 0.033, 0.048), the energy‐temperature coefficients derived from transmission are composition‐independent, while those derived from PL decrease by ≈40% with higher Bi content. This apparent contradiction originates from the thermalized carrier redistribution between the valence band and band‐tail states at elevated temperatures and the intrinsic band‐edge thermal sensitivity in the transmission spectra. The dual‐spectroscopy approach is proven to be an effective method for clarifying the effects of band‐tail states on the thermal sensitivity, and provides valuable guidance for the design of stable GaAsBi optoelectronic devices.

## Graphical abstract

An innovative dual‐spectroscopy approach resolves the debate over GaAsBi's emission temperature sensitivity. By combining temperature‐dependent photoluminescence and transmission spectroscopy, the method decouples the contributions of band‐tail states from the intrinsic band‐edge behavior. This clarifies the origin of conflicting thermal coefficients and provides essential guidance for the design of stable GaAsBi‐based devices.

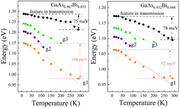

## 1 Introduction

Temperature characteristics critically determine the stability of optoelectronic properties in materials, directly influencing their practical applications.^[^ ^1^, ^2^, ^3^, ^4^ ^]^ Doping is an effective strategy for tuning the optoelectronic properties.^[^ ^5^, ^6^, ^7^, ^8^, ^9^, ^10^, ^11^ ^]^ In this context, ternary semiconductors, such as GaSb_1‐_ *_x_* Bi*_x_* and InP_1‐_ *_x_* Bi*_x_*, have been extensively studied for their temperature‐dependent optical characteristics due to their composition‐tunable electronic properties.^[^ ^12^, ^13^, ^14^, ^15^ ^]^ Research on InPBi revealed unexpected negative thermal quenching in photoluminescence (PL) due to carrier hopping and non‐radiative recombination.^[^ ^14^ ^]^ Based on theoretical models, the effect of temperature on the PL spectra for GaSbBi has been studied.^[^ ^15^ ^]^ Additionally, it was found that hot carrier relaxation in GaSbBi slows down with increasing temperature.^[^ ^6^ ^]^ The contributions of thermal processes to the temperature dependence of the energy gap in dilute Bi III‐V semiconductors, such as InSbBi and InAsBi, have been investigated.^[^ ^16^ ^]^

GaAs_1‐_ *_x_* Bi*_x_*, as a novel semiconductor material, stands out as a strong candidate for long‐wavelength near‐infrared (NIR) optoelectronic devices due to its Bi‐induced significant bandgap reduction and large spin–orbit splitting energy.^[^ ^5^ ^]^ The incorporation of low‐concentration Bi induces a resonant interaction between Bi 6p orbital and the valence band maximum of GaAs,^[^ ^17^ ^]^ leading to significant bandgap reduction and enhanced spin–orbit coupling energy. This mechanism suppresses Auger recombination,^[^ ^18^ ^]^ enhances the superior electron mobility of GaAsBi, and extends carrier lifetime, all of which collectively improve device response speed and efficiency. Bi‐induced band‐tail states in GaAsBi are a hot topic and have been widely investigated, as they critically influence the material's optical and electronic properties. Researchers have conducted some studies on the localized states, such as Zhao et al.’s quantification of the influence of Bi content on localized states,^[^ ^19^ ^]^ Jansson et al.’s finding of surprisingly strong localization persisting to room temperature in GaAs/GaAsBi core/shell nanowires,^[^ ^20^ ^]^ and Dudutiene et al.’s investigation into the effect of barrier growth temperature on the carrier localization.^[^ ^21^ ^]^

However, the effect of Bi on the temperature sensitivity of emission energy remains controversial, despite prior studies exploring the temperature‐dependent optical properties of dilute Bi semiconductors.^[^ ^22^ ^]^ For instance, studies by Oe,^[^ ^23^ ^]^ Fitouri group,^[^ ^24^ ^]^ and Yoshida group^[^ ^25^ ^]^ suggested that higher Bi content reduces the temperature sensitivity, whereas the Kopaczek group^[^ ^26^ ^]^ and Francoeur group^[^ ^27^ ^]^ reported negligible Bi dependence. Therefore, clarifying the cause of the divergent results is an urgent scientific problem that needs to be addressed. While Sarcan et al.’s study describes the evolution of emission involving band‐tail states, its single‐perspective analysis fails to elucidate their participation mechanism.^[^ ^28^ ^]^ Our previous work highlighted the significant role of band‐tail states in dilute bismides.^[^ ^29^ ^]^ Clarifying their influence on the temperature sensitivity of emission energy remains a key challenge. To understand how these states impact the evolution of the energy in GaAsBi, it is therefore imperative to develop a novel, efficient spectroscopic method. PL is an effective technique for probing the optical, thermal, and defect properties of materials.^[^ ^14^, ^15^, ^28^, ^29^, ^30^, ^31^, ^32^ ^]^ Fourier transform infrared (FTIR) spectrometer‐based infrared PL and transmission experiments, benefiting from the high throughput and multiplex advantages of the FTIR spectrometer, have been proven effective in resolving near‐band‐edge electronic structures of narrow‐gap semiconductors such as HgCdTe, InAs/GaSb superlattice, InAsSb and dilute bismuth semiconductors.^[^ ^29^, ^33^, ^34^, ^35^, ^36^ ^]^

Inspired by these, we employ an innovative dual‐spectroscopy approach combining PL and transmission spectroscopy to investigate how band‐tail states impact the temperature sensitivity of GaAsBi emission energy, aiming to elucidate their effect on its temperature‐dependent optoelectronic performance. This work provides an approach to assess band‐tail states, enabling the rational design of thermally stable GaAsBi devices and offering a universal diagnostic toolkit for other disordered semiconductors. High Bi‐composition GaAs_1‐_ *_x_* Bi*_x_* (typically x>0.03^[^ ^37^, ^38^ ^]^) is a suitable candidate for investigating the band‐tail effect due to its significant density of states. Therefore, two 200 nm‐thick GaAs_1‐_ *_x_* Bi*_x_* epitaxial layers with nominal Bi compositions of *x* = 3.3% and 4.8% grown on GaAs substrates by molecular beam epitaxy (MBE) at 354 °C were selected.

## 2 Results

**Figure 1:**
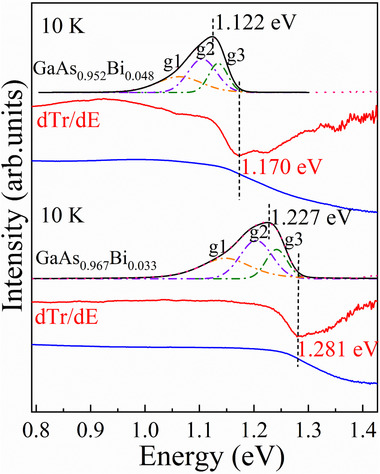
PL spectra with line fittings and transmission spectra with first‐order derivative for GaAs_0.967_Bi_0.033_ and GaAs_0.952_Bi_0.048_ at 10 K.

**Figure** **1** shows representative PL spectra (black curves) for GaAs_0.967_Bi_0.033_ and GaAs_0.952_Bi_0.048_ at 10 K under 80 mW, along with the transmission spectra (blue curves) obtained at the same temperature. To resolve subtle transition features, first‐order derivatives^[^ ^39^ ^]^ are performed on the transmission spectra (red curves) and Gaussian–Lorentzian mixed‐function fittings are applied to the PL spectra (dash‐dot curves). The PL fitting aligns with previous studies, supported by a second‐order derivative analysis.^[^ ^29^ ^]^

The spectral derivatives of the transmission spectra reveal prominent minima at 1.281 eV for GaAs_0.967_Bi_0.033_ and 1.170 eV for GaAs_0.952_Bi_0.048_, marked by vertical dashed lines in Figure 1. These energies closely align with the theoretical bandgap predictions based on the Bi content.^[^ ^40^ ^]^ From these values, a Bi‐induced bandgap reduction rate of 69 meV per 1% Bi incorporation is deduced, consistent with the reported bandgap reduction rate of 60–90 meV/1% Bi in GaAsBi systems.^[^ ^41^ ^]^ It is therefore safe to ascribe the transmission‐derived energies to the fundamental valence‐band to conduction‐band bandedge transitions.

For the PL spectra, i) the PL peak maxima at 1.227 eV (GaAs_0.967_Bi_0.033_) and 1.122 eV (GaAs_0.952_Bi_0.048_), highlighted by dashed lines, demonstrate Bi‐induced bandgap narrowing, which is consistent with the transmission spectral behavior^[^ ^42^ ^]^; ii) these PL peak energies lie systematically below their respective transmission‐derived bandgap values; and iii) the high‐energy PL edges align precisely with transmission‐derived bandedge transition energies, forming a spectral Stokes shift of 54.1 meV (GaAs_0.967_Bi_0.033_) or 47.5 meV (GaAs_0.952_Bi_0.048_) which closely matches the reported values for GaAsBi.^[^ ^43^ ^]^

Notably, GaAs_0.967_Bi_0.033_ exhibits a broader PL linewidth of 308.4 meV, compared to 244.8 meV for GaAs_0.952_Bi_0.048_. PL lineshape is governed by the energy distribution of defect‐related states. An exponential tail model can be used for band‐tail states^[^ ^44^ ^]^:

(1) $$D_{tail}\;\left(E\right)=\frac{N}{w}\;exp\left(\frac{E_{v}-E}{k_{B}T}\right)$$

where ***N*** is the amount of band‐tail localized states, ***w*** is the band‐tail width, ***k_B_*** is the Boltzmann constant with a value of 8.62 × 10^−5^ eV K^−1^, ***T*** is the temperature. This is precisely the case for GaAs_0.952_Bi_0.048_, where the energy distribution of defect states is more concentrated, resulting in a narrower PL lineshape compared to that of GaAs_0.967_Bi_0.033_. This observation aligns with the steeper density of band‐tail states in GaAs_0.952_Bi_0.048_.^[^ ^29^ ^]^ Gaussian–Lorentzian deconvolution reveals three transition features (marked as g1–g3) in each asymmetric PL profile.

**Figure 2:**
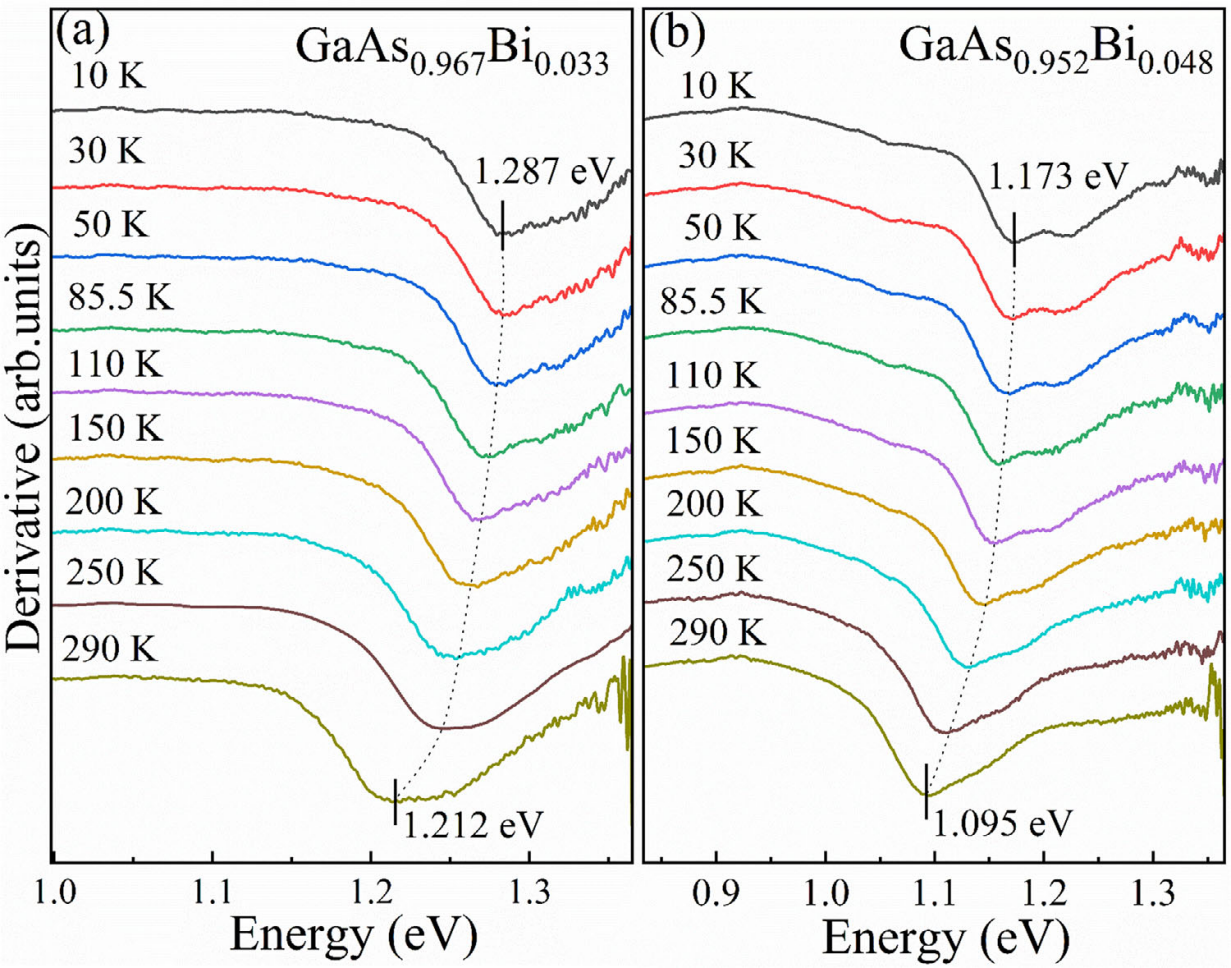
First‐order derivatives of transmission spectra of GaAs_0.967_Bi_0.033_ a) and GaAs_0.952_Bi_0.048_ b) in a temperature range of 10–290 K. The dotted arrows guide the eye to the evolution of the features' energies with temperature.

To investigate the impacts of band‐tail states on thermal behavior, temperature‐dependent PL and transmission spectra were acquired from 10 to 290 K. **Figure** **2** depicts the temperature evolution of transmission derivatives for the GaAs_0.967_Bi_0.033_ and GaAs_0.952_Bi_0.048_ samples. The band‐edge energy decreases by 75 meV (from 1.287 to 1.212 eV) for the GaAs_0.967_Bi_0.033_ and by 78 meV (from 1.173 to 1.095 eV) for the GaAs_0.952_Bi_0.048_ as the temperature rises from 10 to 290 K. Both exhibits nearly identical temperature sensitivity, contrasting sharply with previous reports of Bi‐induced bandgap temperature insensitivity.^[^ ^23^, ^24^, ^25^, ^45^ ^]^

While the intrinsic bandgap states determine the absorption edge position, band‐tail states introduce spectral broadening via localized state transitions. The competition between the bandgap thermal shrinkage and the band‐tail state redistribution creates superposition in the transmission spectra. This is exactly the main reason for the temperature insensitivity that contradicts the prior report.^[^ ^23^, ^24^, ^25^, ^45^ ^]^ The thermal‐induced redistribution of carriers in the relatively steeper band‐tail density of GaAs_0.952_Bi_0.048_,^[^ ^29^ ^]^ results in a more pronounced contribution from tail states in the transmission spectrum, ultimately counteracting the Bi‐induced thermal insensitivity of the bandgap. This leads to comparable apparent temperature sensitivities between the two samples with different Bi content.

**Figure 3:**
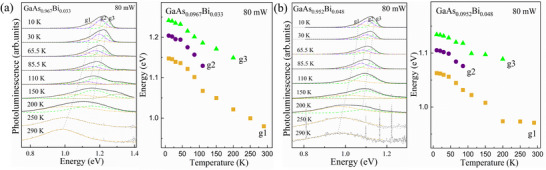
Normalized PL spectra with curve fittings and the corresponding evolution of fitted peaks energy with temperature for GaAs_0.967_Bi_0.033_ a) and GaAs_0.952_Bi_0.048_ b) at excitation powers of 80 mW.

**Figure** **3** illustrates the temperature‐dependent PL spectra for the GaAs_0.967_Bi_0.033_ and GaAs_0.952_Bi_0.048_, measured under an excitation power of 80 mW, as well as the corresponding lineshape fitting results and the energy evolution. As clearly demonstrated, all the three PL fittings (g1–g3) exhibit a redshift with increasing temperature, as tracked by the dot arrows. Previous ***k*·*p*** modeling^[^ ^46^ ^]^ revealed room‐temperature bandgaps of GaAs_1‐_ *_x_* Bi*_x_* as ≈1.15 eV (*x *= 3.3%) and ≈1.07 eV (*x* = 4.8%), which are obviously higher than the experimental PL energies (≈0.98 and ≈0.97 eV, respectively), and hence support the band‐tail assignment. Inhomogeneities in composition and structure lead to the formation of distinct domains at large scales, such as tens of micrometers, which in turn give rise to several distinct band tails.^[^ ^29^, ^47^, ^48^ ^]^ Accordingly, the three features observed in the PL spectra can be attributed to different tail states arising from the inhomogeneities.^[^ ^29^ ^]^ The competing contributions of the three features shape the overall PL profile and its thermal evolution. The g1 features persist at elevated temperatures, while the g2 features quench at 110 K for GaAs_0.967_Bi_0.033_ and at 85.5 K for GaAs_0.952_Bi_0.048_. The g3 features remain detectable up to 200 K. The quenching of the PL emission with increasing temperature is due to thermally activated nonradiative recombination. The thermal quenching of the three fitting PL intensity can be described by^[^ ^49^ ^]^:

(2) $$I\left(T\right)=\frac{I_{0}}{1+\sum_{i}C_{i}e^{-\Delta E_{i}/k_{B}T}}$$

where ***I*_0_** is a saturation intensity, ***C_i_*** reflects the ratio of *i*th nonradiative and radiative recombination rates, ***∆E_i_*** reflects the *i*th activation energy. The quenching order is determined by the value of $C_{i}e^{-\Delta E_{i}/k_{B}T}$, which requires simultaneous consideration of both the ***∆E_i_*** and the ***C_i_***. The high non‐radiative recombination channel density of g2 makes it quench first.

**Figure 4:**
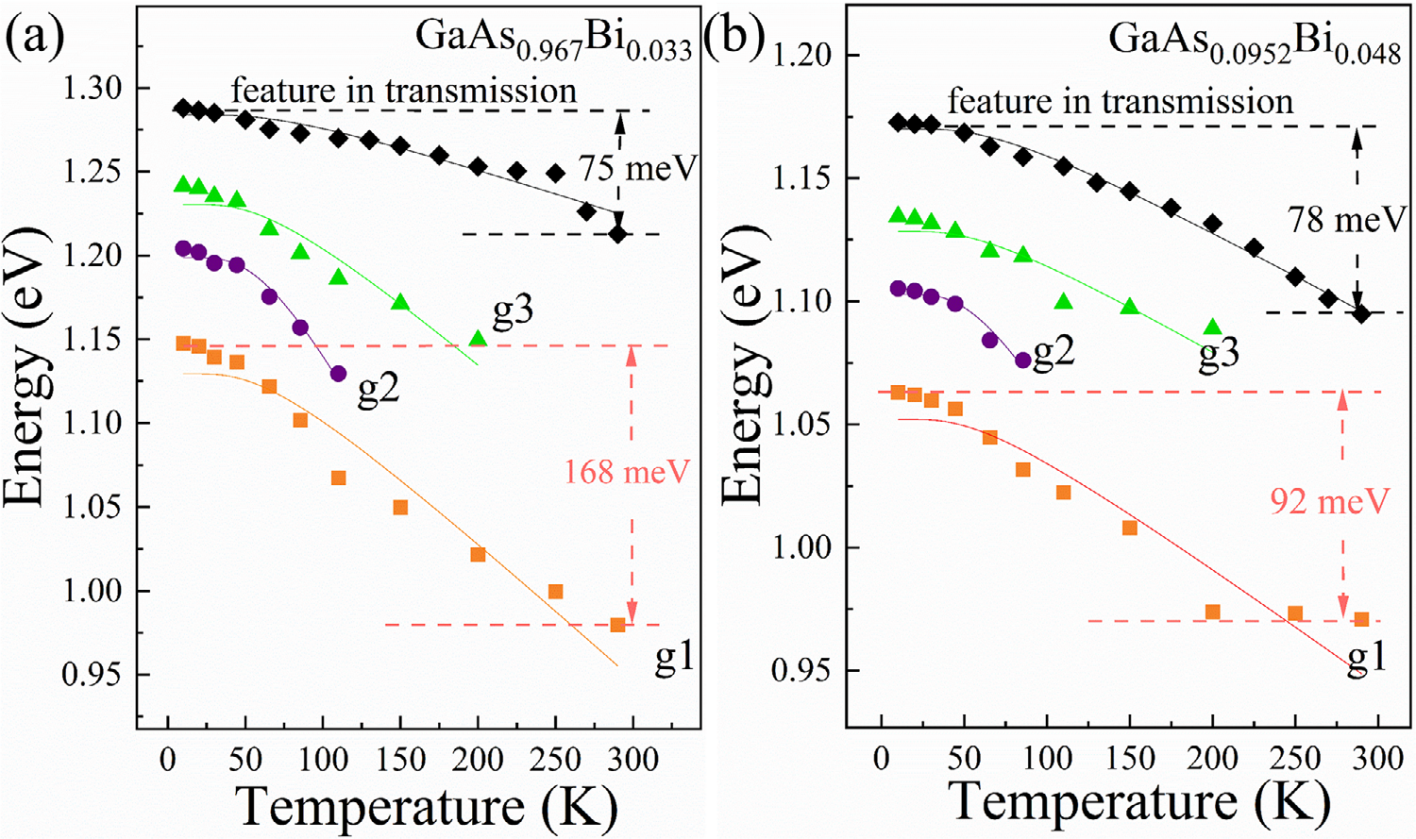
Comparison of the temperature‐dependent evolution of bandedge‐related features (from transmission) and band‐tail states (g1, g2, g3, from PL) for GaAs_0.967_Bi_0.033_ a) and GaAs_0.952_Bi_0.048_ b) under an excitation power of 80 mW.

**Figure** **4** illustrates the comparison of the temperature‐dependent evolution between bandgap‐related features in transmission spectra and band‐tail states features (g1, g2, g3) in the PL spectra for GaAs_0.967_Bi_0.033_ and GaAs_0.952_Bi_0.048_. The g1‐feature presents across the full temperature range, and shows energy redshifts of 168 meV (GaAs_0.967_Bi_0.033_) and 92 meV (GaAs_0.952_Bi_0.048_) as temperature rises from 10 to 290 K. Notably, these energy variations exceed those of the band‐edge transitions by 124% and 18%, respectively, and hence highlight the band‐tail states' dominant role in thermal response. The transition energies vs temperature are plotted, and modeled quantitatively by the following expression derived in a frame of the entropy and enthalpy of electron‐hole pairs formation,^[^ ^50^ ^]^

(3) $$E\;\left(T\right)=E_{0}\;-S\langle \Theta \rangle \left[\coth\left(\frac{\langle \Theta \rangle }{2T}\right)-1\right]$$

where ***E*_0_** is the energy at 0 K, ***S*** is a coupling coefficient describing the electron‐phonon interaction, and **〈Θ〉** is an average phonon temperature set in the fitting process based on the reported value.^[^ ^51^ ^]^ The fittings are directly overlaid on the experimental data as solid lines, with the deduced parameters listed in **Table** **1**.

**Table 1:** The energy (***E*_0_**) at 0 K, coupling coefficient (***S***), and average phonon temperature (**⟨Θ⟩**) derived from the fitting of the energy evolution with temperature. The numbers in parentheses represent the errors in the last digit(s).

	GaAs_0.967_Bi_0.033_					GaAs_0.952_Bi_0.048_		
	g1	g2	g3	Transmission	g1	g2	g3	Transmission
***E*_0_** (eV)	1.130(7)	1.199(2)	1.230(4)	1.284(2)	1.052(7)	1.103(2)	1.128(3)	1.170(0)
***S*** (meV K^−1^)	0.42(4)	0.99(7)	0.40(5)	0.16(2)	0.24(3)	0.69(8)	0.20(3)	0.19(1)
**⟨Θ⟩**(K)	188(24)	208(7)	192(22)	221(21)	176(29)	197(10)	180(27)	209(7)

For both fundamental bandedge (transmission‐derived) and bandtail‐state transitions (g1–g3), the reduction in transition energies (***E*_0_**) from GaAs_0.952_Bi_0.048_ to GaAs_0.967_Bi_0.033_ aligns with Bi‐induced bandgap narrowing. The coupling coefficients ***S*** of the PL transitions (g1–g3 features) in GaAs_0.952_Bi_0.048_ are systematically lower than those in GaAs_0.967_Bi_0.033_, which suggests that i) GaAs_0.952_Bi_0.048_ exhibits a lower temperature sensitivity of the band‐tail states, because higher Bi content introduces steeper band‐tail states^[^ ^29^ ^]^ that provide more carrier trapping sites, thus reducing the extent of the energy position shift with temperature, and ii) enhanced lattice disorders suppress conventional phonon modes and thus reduce electron‐phonon coupling. This disagrees obviously with the transmission‐derived coupling coefficient ***S*** (the latter shows negligible Bi‐content‐induced variation), demonstrating that the role of band‐tail states should be considered for thermal properties of GaAsBi.

**Figure 5:**
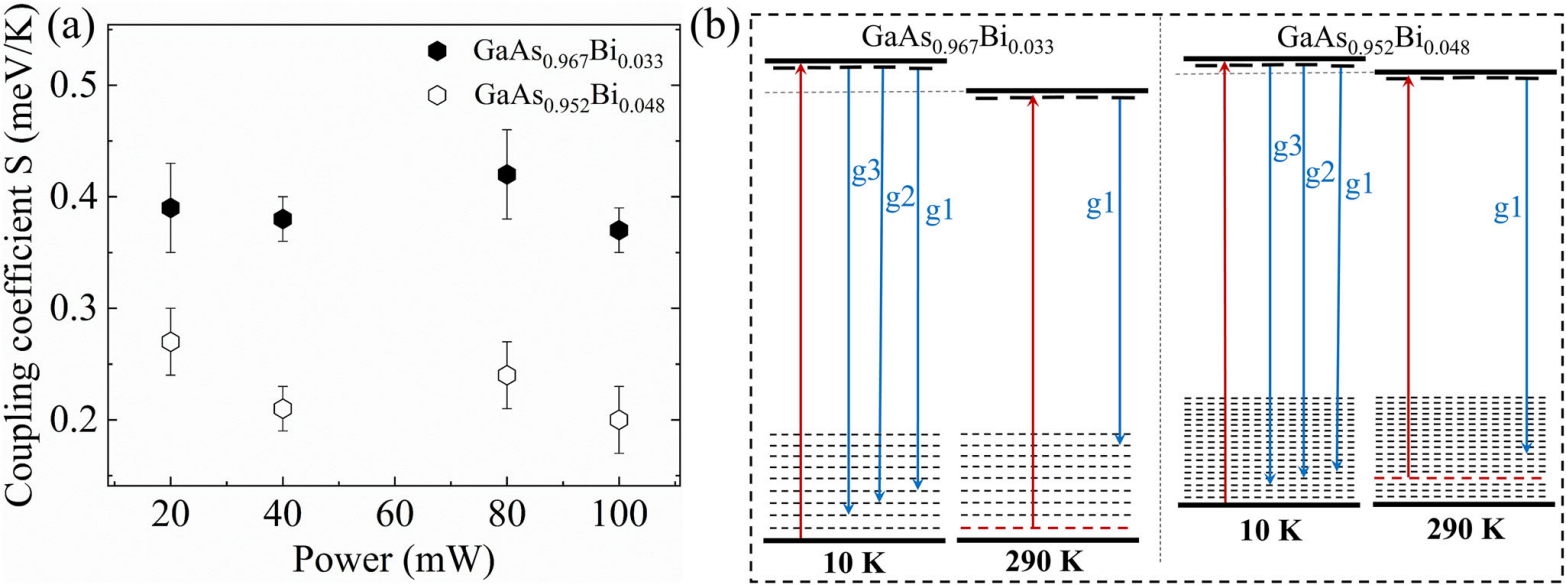
a) The coupling coefficient ***S*** of g1 for GaAs_0.952_Bi_0.048_ and GaAs_0.967_Bi_0.033_, derived from the energy evolution with temperature at several excitation powers. b) Schematic diagram of transmission and PL in GaAs_1‐_ *_x_* Bi*_x_*, with red and blue arrows for the absorption edges and the PL processes, respectively.

The identification of multiple PL transition features enables us to assign a specific PL feature to the transition associated with the band tail. **Figure** **5**a illustrates the main fitting parameters coupling coefficient ***S*** for g1 in GaAs_0.952_Bi_0.048_ and GaAs_0.967_Bi_0.033_. These values were derived from temperature‐dependent energy evolution measurements under multiple excitation powers (see Figure S1, Supporting Information). The power‐dependent results confirm that excitation power does not alter the consistent temperature‐sensitivity trend, with lower coupling coefficients observed in 4.8% Bi samples compared to 3.3% Bi samples. This validates the conclusion regarding the enhanced band‐tail stability in higher Bi‐content GaAs_1‐_ *_x_* Bi*_x_*.

The three PL features reflect radiative recombination of carriers relating to valence band‐tail states and the states below the conduction‐band edge.^[^ ^29^ ^]^ The transmission spectroscopy probes the optical absorption edge, reflecting coupled contributions from band‐edge and band‐tail states. Figure 5b shows a schematic of the transmission and PL processes in GaAs_1‐_ *_x_* Bi*_x_*, with red and blue arrows representing the absorption edge and the PL, respectively. In the low‐temperature PL spectra (e.g., 10 K), all three recombination pathways are active, quenching of the g2 and g3 peaks occurs as the temperature increases, leaving the g1 peak to persist. Due to the relatively low density of band‐tail states in GaAs_0.967_Bi_0.033_, the g1 peak displays a greater energy shift as the temperature rises.^[^ ^29^ ^]^ For the high‐Bi‐content GaAs_0.952_Bi_0.048_, a higher Bi content creates steeper band‐tail states,^[^ ^29^ ^]^ which significantly reduces the energy shift with temperature, resulting in apparent thermal insensitivity of the PL peak energy. By comparison, transmission spectroscopy probes the absorption edge, reflecting contributions from both band‐edge and band‐tail states. These two contributions together determine the energy‐temperature behavior. At low temperatures, such as 10 K, suppressed thermal excitation confines carriers to the valence band, so band‐edge transitions dominate. As temperature increases, carriers are thermally activated into valence band‐tail states, enabling band tail‐to‐conduction transitions. The steeper valence band‐tail density^[^ ^29^ ^]^ in GaAs_0.952_Bi_0.048_ amplifies this consequence of redistribution, making the band‐tail contribution more noticeable in the transmission spectra and thereby counteracting the Bi‐induced insensitivity of the bandgap to temperature. The carrier‐redistribution model reconciles the apparent contradiction between the transmission‐derived temperature sensitivity and the prior reports of Bi‐induced thermal stability.^[^ ^42^ ^]^ In essence, Bi‐related band‐tail states render PL relatively temperature‐insensitive, but their competing contribution to absorption processes can negate the apparent Bi‐induced bandgap thermal stability.

**Table** **2** compares the energy‐temperature coefficients of various III‐V dilute‐Bi semiconductors (GaAs_1‐_ *_x_* Bi*_x_*, InAs_1‐_ *_x_* Bi*_x_*, GaSb_1‐_ *_x_* Bi*_x_*, InSb_1‐_ *_x_* Bi*_x_*), including our representative g1 results. Most compellingly, for the same Bi content *x* in GaAs_1‐_ *_x_* Bi*_x_*, significantly different results have been reported by various research groups. From the perspective of our findings, the discrepancy in the reported temperature sensitivity of energy for GaAs_1‐_ *_x_* Bi*_x_* can be attributed to the varying degrees of contribution from the Bi‐induced band‐tail states, as detected by different methods. A key observation is that for all dilute Bi semiconductors in Table 2, as the Bi content increases, the energy‐temperature coefficient does not decrease monotonically. For example, the energy‐temperature coefficient for GaSb_1‐_ *_x_* Bi*_x_* and InAs_1‐_ *_x_* Bi*_x_* exhibits a fluctuating behavior, and that for InSb_1‐_ *_x_* Bi*_x_* even shows a slight increase. This phenomenon indicates that more than one contribution is at play in dilute Bi semiconductors, specifically the combined effect of bandedge and band‐tail states as proposed in this work, where both act synergistically.

**Table 2:** Energy‐temperature coefficients for dilute Bi semiconductors (GaAs_1‐_ *_x_* Bi*_x_*, GaSb_1‐_ *_x_* Bi*_x_*, InAs_1‐_ *_x_* Bi*_x_*, and InSb_1‐_ *_x_* Bi*_x_*), as reported in the literature, along with our results.

	*x*_Bi_ [%]	Coefficient [meV K^−1^]
GaAs_1‐_ *_x_* Bi*_x_*	0.0	0.54^[^ ^52^ ^]^
	0.2	0.51^[^ ^16^ ^]^
	0.5	0.19^[^ ^25^ ^]^
	1.2	0.40^[^ ^27^ ^]^
	1.3	0.18,^[^ ^25^ ^]^ 0.49^[^ ^16^ ^]^
	2.0	0.41^[^ ^16^ ^]^
	2.6	0.13^[^ ^25^ ^]^
	3.0	0.44^[^ ^53^ ^]^
	3.1	0.40^[^ ^27^ ^]^
	3.7	0.39^[^ ^16^ ^]^
	3.3 (our work)	Bandtail: 0.42, Bandedge: 0.16
	4.8 (our work)	Bandtail: 0.24, Bandedge: 0.19
GaSb_1‐_ *_x_* Bi*_x_*	0.0	0.37^[^ ^26^ ^]^
	0.7	0.39^[^ ^26^ ^]^
	2.1	0.37^[^ ^26^ ^]^
	4.2	0.32^[^ ^26^ ^]^
	6.6	0.51^[^ ^54^ ^]^
	8.3	0.34^[^ ^54^ ^]^
	10.1	0.63^[^ ^54^ ^]^
InAs_1‐_ *_x_* Bi*_x_*	0.0	0.28,^[^ ^55^ ^]^ 0.32,^[^ ^56^ ^]^ 0.26^[^ ^16^ ^]^
	1.8	0.29,^[^ ^56^ ^]^ 0.27^[^ ^16^ ^]^
	2.2	0.27,^[^ ^56^ ^]^ 0.25^[^ ^16^ ^]^
	3.2	0.23,^[^ ^56^ ^]^ 0.22^[^ ^16^ ^]^
	3.7	0.22,^[^ ^56^ ^]^ 0.21^[^ ^16^ ^]^
InSb_1‐_ *_x_* Bi*_x_*	0.0	0.27^[^ ^57^ ^]^
	4.0	0.31^[^ ^16^ ^]^
	5.0	0.32^[^ ^16^ ^]^

The dual‐spectroscopy approach resolves the challenge of distinguishing intrinsic bandedge behavior from extrinsic band‐tail effects in high‐Bi‐composition GaAsBi. PL spectroscopy is sensitive to the band‐tail transitions but can easily miss the intrinsic bandedge ones when the density of band‐tail states is considerably higher than the photo‐induced carrier concentration. Transmission spectroscopy, in principle, reveals the absorption edge. However, it is limited by the insufficient natural resolution for the band tail states. Each method provides an incomplete and potentially misleading view. The advantages of the dual‐spectroscopy approach lie in its integration of PL and transmission data. It leverages the high sensitivity of PL to detect band‐tail activities, while utilizing transmission data to anchor the bandedge. This clarifies the contribution of band tails to thermal sensitivity while providing design principles for temperature‐stable GaAsBi‐based optoelectronics. Furthermore, the approach is a universal diagnostic toolkit for analyzing other semiconductor systems where disorder creates localized states. It can establish a baseline for band‐tail characteristics and track their evolution under varying growth and operational conditions, providing direct feedback for material and device optimization.

## 3 Conclusion

To conclude, this work aimed to resolve the long‐standing debate on Bi content‐dependent emission temperature sensitivity in GaAs_1‐_ *_x_* Bi*_x_* and clarify the unclear role of band‐tail states. To achieve this objective, an innovative dual‐spectroscopy approach (combining temperature‐dependent PL and transmission spectroscopy) is employed. Three band tail‐related transitions are identified in the asymmetric PL peak by Gaussian–Lorentzian fitting. Temperature‐dependent PL results reveal lower temperature sensitivity of band tail states in higher Bi‐contented GaAs_1‐_ *_x_* Bi*_x_*, with PL‐derived energy‐temperature coefficients decreasing by ≈40% from *x* = 0.033 to 0.048. However, the energy‐temperature coupling coefficient derived from transmission spectra exhibits Bi‐content‐independent property. This discrepancy indicates that prior debated Bi‐induced variations in the temperature sensitivity are governed by thermal carrier redistribution between the valence band and band‐tail states. The study resolves the ambiguity surrounding the impact of band‐tail states on GaAsBi's thermal emission, identifies band‐tail engineering as a key design parameter, highlights the need for accurate carrier dynamics modeling, and provides a practical validation method to guide material temperature‐sensitivity assessment.

## 4 Experimental Section

A DCA P600 MBE reactor, which was equipped with high‐purity elemental sources (Ga: 99.999%, As: 99.999% as As_2_, Bi: 99.999%), was used for growing two 200 nm GaAs_1‐_ *_x_* Bi*_x_* epilayers on GaAs substrates. The growth temperature of the GaAs_1‐_ *_x_* Bi*_x_* was 354 °C read by a thermocouple. Ga beam equivalent pressure (BEP) was fixed at 2.2 × 10^−7^ Torr, corresponding to a growth rate of 0.4 ML s^−1^. The As_2_ BEP was adjusted on the brink of As shortage to incorporate Bi into the epilayer, namely between 8.2 × 10^−7^ to 9.4 × 10^−7^ Torr. The BEP ratio of As_2_/Ga was close to 4. Bi content in the GaAs_1‐_ *_x_* Bi*_x_* was varied by changing Bi to As BEP ratio, and was estimated to be 3.3% and 4.8%, respectively, with X‐ray diffraction (XRD) data.^[^ ^29^ ^]^

Temperature‐dependent PL and transmission spectra were acquired using a multiple‐function FTIR spectroscopy system,^[^ ^58^, ^59^ ^]^ at a spectral resolution of 12 cm^−1^, sufficient to resolve multiple electronic transition processes near the band edge. Samples were mounted in a liquid‐helium continuous‐flow cryostat enabling precise temperature control from 10 to 290 K. A 532 nm excitation laser offers the power of 20 to 100 mW in a spot with diameter of ≈100 µm, which ensures negligible laser heating effect. Its penetration depth covers the entire GaAsBi thickness, integrating PL signals over a broader volume encompassing various structural features.

## Conflict of Interest

The authors declare no conflict of interest.

## Supporting information



Supporting Information

## Data Availability

The data that support the findings of this study are available from the corresponding author upon reasonable request.

## References

[1] S. Yu , J. Xu , X. Shang , E. Ma , F. Lin , W. Zheng , D. Tu , R. Li , X. Chen , Adv. Sci. 2021, 8, 2100084. 10.1002/advs.202100084 PMC8498867 34382362

[2] Y. Huang , Z. Lai , J. Jin , F. Lin , F. Li , L. Lin , D. Tian , Y. Wang , R. Xie , X. Chen , Small 2021, 17, 2103425. 10.1002/smll.202103425 34647396

[3] R. K. Guntu , M. Gopikrishna , S. S. Devi , M. Tejaswi , S. Babu , M. Israr , Appl. Phys. A 2025, 131, 518.

[4] R. K. Guntu , J. Mol. Struct. 2022, 1248, 131533.

[5] Y. Liu , X. Yi , N. J. Bailey , Z. Zhou , T. B. O. Rockett , L. W. Lim , C. H. Tan , R. D. Richards , J. P. R. David , Nat. Commun. 2021, 12, 4784. 34362898 10.1038/s41467-021-24966-0 PMC8346614

[6] A. S. Sharma , S. J. Sreerag , R. N. Kini , J. Appl. Phys. 2024, 135, 035701.

[7] Z. Yang , M. Wang , D. Yu , L. Zhu , J. Shao , X. Chen , J. Infrared Millim. Waves 2023, 42, 730.

[8] N. R. K. Chand , J. Budida , C. S. Rao , R. K. Guntu , Appl. Phys. A 2025, 131, 532.

[9] K. Ashok , N. Yuganand , R. K. Guntu , E. D. Francis , Radiat. Phys. Chem. 2024, 224, 112057.

[10] K. V. Rao , M. Madhu , P. Ashok , G. A. Kumar , R. K. Guntu , Silicon 2022, 14, 9887.

[11] B. K. Sudhakar , N. R. K. Chand , V. Tirupati , S. PVS , G. R. Kumar , G. S. Rao , C. S. Rao , Phys. Scr. 2023, 98, 065926.

[12] L. Yue , X. Chen , Y. Zhang , F. Zhang , L. Wang , J. Shao , S. Wang , J. Alloy Comp. 2018, 742, 780.

[13] O. Delorme , L. Cerutti , E. Luna , G. Narcy , A. Trampert , E. Tournié , J.‐B. Rodriguez , Appl. Phys. Lett. 2017,110, 222106.

[14] X. Chen , X. Wu , L. Yue , L. Zhu , W. Pan , Z. Qi , S. Wang , J. Shao , Appl. Phys. Lett. 2017, 110, 051903.

[15] T. Hidouri , I. Mal , D. P. Samajdar , F. Saidi , T. D. Das , Superlattices Microstruct. 2019, 129, 252.

[16] S. Zouaghi , H. Fitouri , A. Rebey , Solid State Commun. 2022, 343, 114649.

[17] B. Mondal , M. Kröner , T. Hepp , K. Volz , R. Tonner‐Zech , Phys. Rev. B 2023, 108, 035202.

[18] Y. Kunihashi , Y. Shinohara , S. Hasegawa , H. Nishinaka , M. Yoshimoto , K. Oguri , H. Gotoh , M. Kohda , J. Nitta , H. Sanada , Appl. Phys. Lett. 2023, 122, 182402.

[19] C. Zhao , M. Zhu , J. Wang , S. Wang , K. Lu , Superlattices Microstruct. 2018, 117, 515.

[20] M. Jansson , S. Hiura , J. Takayama , A. Murayama , F. Ishikawa , W. Chen , I. Buyanova , J. Phys. Chem. C 2025, 129, 4456. 10.1039/d4cp03973d 39790050

[21] E. Dudutiene , A. Jasinskas , S. Stanionyte , M. Skapas , A. Vaitkevicius , B. Cechavicius , R. Butkute , Mater. Sci. Semicond. Process 2025, 199, 109828.

[22] W. M. Linhart , R. Kudrawiec , Semicond. Sci. Technol. 2018, 33, 073001.

[23] K. Oe , Jpn. J. Appl. Phys. 2002, 41, 2801.

[24] H. Fitouri , I. Moussa , A. Rebey , B. E. Jani , Microelectron. Eng. 2011, 88, 476.

[25] J. Yoshida , T. Kita , O. Wada , K. Oe , Jpn. J. Appl. Phys. 2003, 42, 371.

[26] J. Kopaczek , R. Kudrawiec , W. M. Linhart , M. K. Rajpalke , K. M. Yu , T. S. Jones , M. J. Ashwin , J. Misiewicz , T. D. Veal , Appl. Phys. Lett. 2013, 103, 261907.

[27] S. Francoeur , M.‐J. Seong , A. Mascarenhas , S. Tixier , M. Adamcyk , T. Tiedje , Appl. Phys. Lett. 2003, 82, 3874.

[28] F. Sarcan , Ö. Dönmez , K. Kara , A. Erol , E. Akalın , M. Ç. Arıkan , H. Makhloufi , A. Arnoult , C. Fontaine , Nanoscale Res. Lett. 2014, 9, 119. 24629075 10.1186/1556-276X-9-119 PMC3984679

[29] B. Yan , X. Chen , L. Zhu , W. Pan , L. Wang , L. Yue , X. Zhang , L. Han , F. Liu , S. Wang , J. Shao , Appl. Phys. Lett. 2019, 114, 052104.

[30] R. K. Guntu , Ceram. Int. 2025, 51, 16524.

[31] G. R. Kumar , M. K. Rao , T. Srikumar , M. C. Rao , V. R. Kumar , N. Veeraiah , C. S. Rao , J. Alloys Compd. 2018, 752, 179.

[32] R. K. Guntu , Opt. Quant. Electron. 2024, 56, 255.

[33] J. Shao , L. Chen , F. Zha , W. Lu , X. Lü , S. Guo , L. He , J. Chu , J. Appl. Phys. 2010, 108, 023518.

[34] Q. Zhuang , H. Alradhi , Z. Jin , X. Chen , J. Shao , X. Chen , A. M. Sanchez , Y. Cao , J. Liu , P. Yates , K. Durose , C. Jin , Nanotechnology 2017, 28, 105710. 28177930 10.1088/1361-6528/aa59c5

[35] X. Chen , Z. Xu , Y. Zhou , L. Zhu , J. Chen , J. Shao , Appl. Phys. Lett. 2020, 117, 081104.

[36] J. Shao , Z. Qi , H. Zhao , L. Zhu , Y. Song , X. Chen , F. Zha , S. Guo , S. Wang , J. Appl. Phys. 2015, 118, 165305.

[37] C. Goletti , L. Fazi , E. Tisbi , B. Bonanni , E. Placidi , F. Arciprete , Appl. Phys. Lett. 2022, 120, 031902.

[38] S. Alhassan , D. d. Souza , A. Alhassni , A. Almunyif , S. Alotaibi , A. Almalki , M. Alhuwayz , I. P. Kazakov , A. V. Klekovkin , V. I. Tsekhosh , I. A. Likhachev , E. M. Pashaev , S. Souto , Y. G. Gobato , N. A. Saqri , H. V. A. Galeti , F. Al mashary , H. Albalawi , N. Alwadai , M. Henini , J. Alloys Compd. 2021, 885, 161019.

[39] A. Varghese , L. George , Spectrochim. Acta A: Mol. Biomol. Spectrosc. 2012, 95, 46. 22613123 10.1016/j.saa.2012.04.092

[40] Y. Tominaga , K. Oe , M. Yoshimoto , Phys. Status Solidi C 2010, 8, 260.

[41] K. K. Nagaraja , Y. A. Mityagin , M. P. Telenkov , I. P. Kazakov , Crit. Rev. Solid State Mater. Sci. 2016, 42, 239.

[42] E. Tisbi , E. Placidi , R. Magri , P. Prosposito , R. Francini , A. Zaganelli , S. Cecchi , E. Zallo , R. Calarco , E. Luna , J. Honolka , M. Vondrácek , S. Colonna , F. Arciprete , Phys. Rev. Appl. 2020, 14, 014028.

[43] S. Imhof , A. Thränhardt , A. Chernikov , M. Koch , N. S. Köster , K. Kolata , S. Chatterjee , S. W. Koch , X. Lu , S. R. Johnson , D. A. Beaton , T. Tiedje , O. Rubel , Appl. Phys. Lett. 2010, 96, 131115.

[44] H. Wang , Z. Ji , S. Qu , G. Wang , Y. Jiang , B. Liu , X. Xu , H. Mino , Opt. Express 2012, 20, 3932. 22418149 10.1364/OE.20.003932

[45] M. Fregolent , M. Buffolo , C. D. Santi , S. Hasegawa , J. Matsumura , H. Nishinaka , M. Yoshimoto , G. Meneghesso , E. Zanoni , M. Meneghini , J. Phys. D: Appl. Phys. 2021, 54, 345109.

[46] H. Li , Z. M. Wang , in Bismuth‐containing compounds, Springer, London, UK 2013.

[47] J. Shao , M. Wang , X. Chen , L. Zhu , F. Zha , H. Zhao , S. Wang , W. Lu , Chin. Phys. B 2025, 34, 107802.

[48] X. Chen , M. Wang , L. Zhu , H. Xie , L. Chen , J. Shao , Appl. Phys. Lett. 2023, 123, 151105.

[49] X. Chen , Y. Zhou , L. Zhu , Z. Qi , Q. Xu , Z. Xu , S. Guo , J. Chen , L. He , J. Shao , Jpn. J. Appl. Phys. 2014, 53, 082201.

[50] X. Chen , Q. Zhuang , H. Alradhi , Z. Jin , L. Zhu , X. Chen , J. Shao , Nano Lett. 2017, 17, 1545. 28231002 10.1021/acs.nanolett.6b04629

[51] C. Sergio , C. Duarte , C. Anzola , G. de Aquino , G. Gusev , J. Lumin. 2018, 202, 322.

[52] I. Vurgaftman , J. R. Meyer , L. R. Ram‐Mohan , J. Appl. Phys. 2001, 89, 5815.

[53] A. R. Mohmad , F. Bastiman , C. J. Hunter , R. D. Richards , S. J. Sweeney , J. S. Ng , J. P. R. David , B. Y. Majlis , Phys. Status Solidi B 2014, 251, 1276.

[54] L. Yue , X. Chen , Y. Zhang , J. Kopaczek , J. Shao , M. Gladysiewicz , R. Kudrawiec , X. Ou , S. Wang , Opt. Mater. Express 2018, 8, 893.

[55] A. Boutramine , J. Electron. Mater. 2023, 52, 6031.

[56] H. Okamoto , K. OE , Jpn. J. Appl. Phys. 1999, 38, 1022.

[57] P. Y. Liu , J. C. Maan , Phys. Rev. B 1993, 47, 16274. 10.1103/physrevb.47.16274 10006052

[58] L. Zhu , J. Shao , X. Chen , Y. Li , L. Zhu , Z. Qi , T. Lin , W. Bai , X. Tang , J. Chu , Phys. Rev. B 2016, 94, 155201.

[59] J. Shao , X. Chen , M. Wang , W. Lu , Acta Phys. Sin. 2025, 74, 017801.

