# Supplementary material for: Unraveling Band‐Tail Effects on Temperature‐Dependent Emission in GaAsBi via Photoluminescence
Source: Adv Sci (Weinh). 2025 Nov 30;13(9):e16349. doi: 10.1002/advs.202516349 (PMC12904009; doi:10.1002/advs.202516349)
Supplement: Supplementary file 1 — Supporting Information [file ADVS-13-e16349-s001.docx]

Supporting Information

Unraveling Band-Tail Effects on Temperature-Dependent Emission in GaAsBi via Photoluminescence

Bing Yan*, Xiren Chen*, Liangqing Zhu, Lijuan Wang, Man Wang, Shumin Wang, and Jun Shao*

**Figure S1** shows the temperature-dependent photoluminescence (PL) spectra for the GaAs_0.967_Bi_0.033_ and GaAs_0.952_Bi_0.048_ samples measured under various excitation powers of 20 mW, 40 mW, and 100 mW. The corresponding lineshape fitting results and the evolution of the peak energies are also shown. These results are consistent with the data presented in Figure 3 (main text) measured at 80 mW, showing a similar red shift of all PL components (g1-g3) with increasing temperature. Those results complement the analysis presented in Figure 5 of the main text. It confirms that the observed temperature-dependent red shift of the PL peaks and the trend of coupling coefficient *S* with Bi composition are robust and not dependent on the specific excitation power used.

**Figure S1**. Normalized PL spectra with curve fittings and the corresponding evolution of fitted peaks energy with temperature for GaAs_0.967_Bi_0.033_ (a) and GaAs_0.952_Bi_0.048_ (b) at excitation powers of 20 mW, 40 mW, and 100 mW, respectively.
